# Advancing cyberbullying detection in low-resource languages: a transformer- stacking framework for Bengali

**DOI:** 10.3389/frai.2025.1679962

**Published:** 2026-01-13

**Authors:** Md. Nesarul Hoque, Rudra Pratap Deb Nath, Abu Nowshed Chy, Debasish Ghose, Md Hanif Seddiqui

**Affiliations:** 1 Big Data, Information and Knowledge Engineering Lab, Department of Computer Science and Engineering, University of Chittagong, Chattogram, Bangladesh; 2 Department of Computer Science and Engineering, Gopalganj Science and Technology University, Gopalganj, Bangladesh; 3 School of Economics, Innovation and Technology, Kristiania University of Applied Sciences, Bergen, Norway; 4 The Computational Modeling Group, University of Cambridge, Cambridge, United Kingdom

**Keywords:** additional preprocessing, Bengali, cyberbullying, low-resource language, transformer integration

## Abstract

Cyberbullying on social networks has emerged as a pressing global issue, yet research in low-resource languages such as Bengali remains underdeveloped due to the scarcity of high-quality datasets, linguistic resources, and targeted methodologies. Many existing approaches overlook essential language-specific preprocessing, neglect the integration of advanced transformer-based models, and do not adequately address model validation, scalability, and adaptability. To address these limitations, this study introduces three Bengali-specific preprocessing strategies to enhance feature representation. It then proposes *Transformer-stacking*, an effective hybrid detection framework that combines three transformer models, XLM-R-base, multilingual BERT, and Bangla-Bert-Base, via a stacking strategy with a multi-layer perceptron classifier. The framework is evaluated on a publicly available Bengali cyberbullying dataset comprising 44,001 samples across both binary (*Sub-task A*) and multiclass (*Sub-task B*) classification settings. *Transformer-stacking* achieves an F1-score of 93.61% and an accuracy of 93.62% for *Sub-task A*, and an F1-score and accuracy of 89.23% for *Sub-task B*, outperforming eight baseline transformer models, four transformer ensemble techniques, and recent state-of-the-art methods. These improvements are statistically validated using McNemar's test. Furthermore, experiments on two external Bengali datasets, focused on hate speech and abusive language, demonstrate the model's scalability and adaptability. Overall, *Transformer-stacking* offers an effective and generalizable solution for Bengali cyberbullying detection, establishing a new benchmark in this underexplored domain.

## 1 Introduction

Social media platforms such as Facebook and X (formerly Twitter) have become powerful tools for sharing opinions and sentiments. However, the open nature of these platforms has also led to the proliferation of harmful content, including cyberbullying. The anonymity afforded by social networks often emboldens individuals to engage in harmful behavior without facing immediate consequences (Kim et al., 2023). The frequency and severity of cyberbullying incidents have increased in recent years, particularly during the COVID-19 pandemic (Kee et al., 2022). Research links cyberbullying to psychological effects such as anxiety, depression, low self-esteem, and suicidal ideation, along with social isolation and long-term trauma (Peled, 2019). In some cases, bullying messages, even those circulated online, can trigger religious or communal unrest in real-world settings, particularly when amplified by rumor and hate speech via social media platforms (Roy et al., 2023). Therefore, an automatic detection and analysis of cyberbullying content is essential to mitigate its impact (Jacobs et al., 2020). Swift identification enables authorities to respond quickly and supports the tracking of responsible individuals. Despite its importance, this task remains complex, especially in the presence of informal language, sarcasm, and implicit abuse (Tasnim and Nath, 2024).

Most cyberbullying detection research has focused on the English language (Mishra et al., 2024). Several studies have employed BERT-based hybrid techniques to classify texts as either bullying or non-bullying (Samee et al., 2023; Mali et al., 2025). One approach introduces an online bully-checking system utilizing DistilBERT, a lightweight variant of BERT (Teng and Varathan, 2023). Another method aims to minimize detection latency by combining machine learning (ML) and deep learning (DL) models (Nitya Harshitha et al., 2024). Additionally, Long Short-Term Memory (LSTM) networks have been leveraged to assess the severity level of cyberbullying content. Some research further investigates the classification of specific abuse types, such as religious, ethnic, or gender-based harassment, using a range of ML and DL techniques (Alqahtani and Ilyas, 2024; Jaradat et al., 2025).

In contrast to high-resource languages, Bengali, a low-resource language, has received limited attention in cyberbullying research, primarily due to insufficient preprocessing, high semantic complexity, inherent model limitations, and a lack of validation and robustness (Hoque et al., 2023). Most existing studies rely on general preprocessing steps, such as removing HTML tags, URLs, and punctuation (Sifath et al., 2024; Ghosh and Senapati, 2024), but fail to consider symbolic and contextual cues that are highly relevant in Bengali social media texts. For example, a post such as “
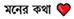
” (Thoughts of the mind 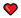) carries a positive, non-bullying sentiment due to the heart emoji, whereas censored expressions like “
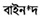
” (bas*tard) are typically used in sexually dehumanizing or abusive contexts. Discarding these symbols during preprocessing removes important signals that can distinguish bullying from non-bullying content. This absence of enriched preprocessing cascades into deeper semantic challenges. Traditional ML and DL models (e.g., LR, SVM, RNNs) mainly rely on surface-level features such as TF-IDF, word counts, or n-grams (Akhter et al., 2023; Hamid et al., 2023; Sifath et al., 2024; Eilertsen et al., 2019) or rule-based (Nath et al., 2025), and thus often miss implicit or context-sensitive forms of bullying. For instance, the text “
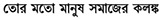
” (People like you are a disgrace to society) carries implicit offense embedded in tone rather than in explicit keywords, which shallow models frequently misclassify as neutral. Recent studies using single transformers, such as multilingual BERT (mBERT) (Aurpa et al., 2022; Hoque and Seddiqui, 2024a), offer stronger contextual representation. However, individual architectures vary in training corpora, tokenization, and optimization, leading to generalization problems. For example, Bangla-Bert-Base, trained primarily on standard Bengali, often struggles with informal or dialect-rich expressions like “
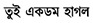
” (You are completely mad/crazy), where non-standard grammar or spelling shifts the sentiment. Moreover, single transformers may develop bias toward specific labels, misclassifying sarcasm or subtle bullying as non-bullying behavior. Thus, combining multiple transformers with complementary strengths (e.g., XLM-R's robust cross-lingual generalization from large-scale training, mBERT's multilingual transfer through shared subword representations, and Bangla-Bert-Base's domain-specific adaptation) remains an underexplored direction.

Even when hybrid or ensemble approaches are introduced, critical aspects of validation and scalability are often overlooked. Many studies do not provide statistical testing, such as McNemar's test, to confirm whether improvements are significant compared to baselines. Similarly, scalability and adaptability remain largely untested, as models are rarely applied to corpora beyond cyberbullying, such as hate speech or abusive language datasets, leaving questions about robustness unanswered.

From this analysis, three key research gaps (RGs) are identified:

RG1 **Insufficient preprocessing:** Inadequate handling of Bengali-specific symbolic and contextual cues in preprocessing.
RG2 **Semantic complexity and model limitations:** Over-reliance on shallow features or single transformer models, limiting the ability to capture semantic complexity.
RG3 **Lack of validation and robustness:** Limited attention to rigorous model validation, adaptability, and scalability across datasets.

These gaps are directly relevant to both binary (*Sub-task A*) and multiclass (*Sub-task B*) classification. In *Sub-task A*, Bengali-specific cues (e.g., emojis, censored terms) enhance discrimination between bullying and non-bullying texts, while advanced semantic modeling and rigorous validation help mitigate misclassification of implicit or sarcastic bullying. In *Sub-task B*, the challenges become more pronounced: symbolic cues often indicate specific bullying categories, semantic nuances are crucial for distinguishing closely related classes (e.g., threat vs. sexual harassment), and robust validation ensures balanced performance across multiple labels. Therefore, addressing the identified gaps is essential for improving both binary and multiclass Bengali cyberbullying detection. To this end, the present study leverages multiple Bengali corpora, applies enriched preprocessing, integrates state-of-the-art (SOTA) transformer models, and conducts systematic validation and robustness checks to enhance classification performance. The main research contributions (RCs), each aligned with the corresponding research gap, are outlined below.

RC1 **Bengali-specific preprocessing enhancements:** We propose and implement three targeted preprocessing strategies to enrich feature representation: (i) replacing censored or unuttered terms (e.g., “
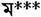
”) with standardized Bengali tokens, (ii) mapping emoticons and emojis to generalized Bengali sentiment expressions, and (iii) injecting class-specific feature terms to improve semantic relevance.
RC2 **Transformer-stacking framework:** We introduce *Transformer-stacking*, a hybrid architecture that integrates three transformer-based models: XLM-R-base, mBERT, and Bangla-Bert-Base, combined with a multi-layer perceptron (MLP) as the meta-classifier. The framework outperforms eight standalone transformer baselines and four ensemble methods, achieving an F1-score of 93.61% and accuracy of 93.62% in *Sub-task A*, and both F1-score and accuracy of 89.23% in *Sub-task B*. In comparative analysis with recent SOTA approaches, including BERT-base (Aurpa et al., 2022), a multi-feature transformer-based deep learning model (Wahid and Al Imran, 2023), XLM-R-base (Emon et al., 2022), and ensemble methods using hard and soft voting (Hoque and Seddiqui, 2023, 2024b), our framework demonstrates consistent superiority, with a 5.69% accuracy improvement in *Sub-task A* and accuracy gains ranging from 1.85% to 4.97% in *Sub-task B* on the widely adopted Bengali cyberbullying dataset (Ahmed et al., 2021a).
RC3 **Rigorous validation and robustness testing:** We conduct extensive experiments to evaluate the proposed framework through: (i) statistical validation using McNemar's test to assess the significance of improvements over eight individual transformer models, and (ii) generalizability testing on two external Bengali datasets (hate speech and abusive language) to assess scalability and adaptability.

The remainder of the paper is structured as follows. Section 2 reviews related work and highlights key limitations. Section 3 elucidates the proposed Transformer-stacking framework. Section 4 details the experimental setup, empirical results, and key insights. Finally, Section 5 presents the conclusion and future research directions.

## 2 Related work

This section provides a comprehensive review of the literature on cyberbullying research, with a specific focus on the classification of textual cyberbullying. Explores the advancements made in high-resource languages, especially English, highlighting the evolution of machine learning and deep learning models for cyberbullying classification. The section also focuses on emerging studies in low-resource languages such as Bengali, where unique linguistic and cultural challenges persist.

### 2.1 Cyberbullying research in high-resource languages (English)

Extensive research on cyberbullying detection has been conducted in high-resource languages, particularly English (Mishra et al., 2024). Most studies focus on binary classification of bullying versus non-bullying content. Samee et al. (2023) proposed a hybrid framework combining emotional features, word2vec embeddings, and federated learning with BERT, achieving 92.15% accuracy while enhancing privacy and robustness. Mali et al. (2025) integrated Binary Chimp Optimization-based Feature Selection technique, Stacked Bidirectional Gated Recurrent Unit Attention, and BERT, yielding 99.12% accuracy. Teng and Varathan (2023) incorporated psycholinguistic and toxicity features with traditional ML models, where fine-tuned DistilBERT achieved 97.41% accuracy and was deployed as an online detection system.

Efficiency-focused (time and accuracy) work by Nitya Harshitha et al. (2024) combined CNN with Random Forest, attaining 95.86% accuracy and a 3.4 × faster runtime than CNN alone. Obaid et al. (2023) extended detection to severity classification (low, medium, high) using LSTM and fuzzy logic, achieving 93.67% accuracy. For fine-grained categorization, Alqahtani and Ilyas (2024) used a stacking ensemble (RF, DT, XGBoost) with TF-IDF bigrams to reach 90.71%, while Jaradat et al. (2025) obtained 91% using BiLSTM on the same corpus.

Beyond English, Alsuwaylimi and Alenezi (2025) developed an Arabic cyberbullying dataset and applied a hybrid CAMelBERT'AraGPT2 model with feature fusion for detection.

Inspired by these resource-rich studies, which largely rely on standalone or hybrid transformer architectures (Mali et al., 2025; Alsuwaylimi and Alenezi, 2025; Samee et al., 2023; Teng and Varathan, 2023), the present research focuses on Bengali, a low-resource language, by integrating advanced preprocessing with hybrid transformer models to enhance class-specific performance.

### 2.2 Cyberbullying research in low-resource languages (Bengali)

Cyberbullying research in Bengali, a low-resource language, remains limited compared to high-resource counterparts. Table 1 summarizes the major studies, comparing them across several key dimensions: Study (citation), Year (publication), Context, Classification Type, AP (additional preprocessing), THF (transformer-based hybrid framework), ST (statistical testing), Sc (scalability), and Ad (adaptability). To align with the identified research gaps (Section 1), these features are organized as follows: RG1—Insufficient preprocessing (AP); RG2—Semantic complexity and model limitations (THF); and RG3—Lack of validation and robustness (ST, Sc, Ad). A detailed review of each Bengali cyberbullying classification study is presented in the subsequent discussion.

**Table 1:** Comparison of existing studies in Bengali cyberbullying research.

**Study**	**Year**	**Context**	**Classification type**	**RG1**	**RG2**	**RG3**		
				**AP**	**THF**	**ST**	**Sc**	**Ad**
Hoque and Seddiqui (2024a)	2024	Threat and abusive	Binary	Yes	No	No	No	No
Hamid et al. (2023)	2023	Slang language	Binary	No	No	No	No	No
Aurpa et al. (2022)	2022	Cyberbullying	Multiclass	No	No	No	No	Yes
Wahid and Al Imran (2023)	2023	Cyberbullying	Multiclass	Yes	Yes	No	No	No
Akhter et al. (2023)	2023	Cyberbullying	Binary and multiclass	No	No	No	No	No
Mohi Uddin et al. (2024)	2024	Cyberbullying	Multiclass	No	No	No	No	No
Sifath et al. (2024)	2024	Cyberbullying	Multiclass	No	No	No	No	No
Islam et al. (2024)	2024	Hate speech	Binary and multiclass	No	No	No	No	No
Nandi et al. (2024)	2024	Hate speech	Binary	No	Yes	No	Yes	Yes
Ghosh and Senapati (2024)	2024	Hate speech	Binary	No	No	No	No	No
Ranasinghe and Zampieri (2021)	2021	Agressive	Multiclass	No	Yes	No	No	Yes
**Our study**	**2025**	**Cyberbullying**	**Binary and multiclass**	**Yes**	**Yes**	**Yes**	**Yes**	**Yes**

Most studies on Bengali cyberbullying detection focus on binary classification using small datasets (typically under 10K samples). Hoque and Seddiqui (2024a) systematically examined data preparation and feature extraction with ML, DL, and transformer models, in which mBERT achieving the best accuracy (80.17%). However, they did not explore hybrid transformers (RG2) or validation strategies (RG3). Hamid et al. (2023) applied TF-IDF with LR and SVM for slang detection (≈70% accuracy) but lacked advanced preprocessing (RG1), hybrid modeling (RG2), and validation (RG3).

For multiclass classification, several works used the dataset from Ahmed et al. (2021a) containing 44K samples across five categories (*Sexual, Troll, Religious, Threat*, and *Not Bully*). Aurpa et al. (2022) fine-tuned mBERT and ELECTRA, achieving 85.00% and 84.92% accuracy, respectively, and demonstrated model adaptability across datasets but omitted validation (RG3). Wahid and Al Imran (2023) proposed a hybrid BERT-based gating mechanism combining contextual, lexical, and social features, yielding 86.30% accuracy, yet did not address robustness or scalability (RG3).

Akhter et al. (2023) applied Instance Hardness Thresholding (IHT) for imbalance reduction, followed by TF-IDF with LR and MLP, reporting 98.57% (binary) and 98.82% (multiclass) accuracy. However, challanging sample filtering and extensive data removal (from 44K to 8.4K samples) undermined result reliability, and RG1–RG3 remained unaddressed. Mohi Uddin et al. (2024) extended this approach by merging datasets and employing a hybrid ensemble (SGD-MLP-LR), achieving 99%+ accuracy, yet with similar limitations regarding preprocessing (RG1), transformer integration (RG2), and robustness (RG3). Sifath et al. (2024) compared RNN, Tri-RNN fusion, and CNN-LSTM-RNN architectures (≈85%–86% accuracy) without tackling any RGs.

Islam et al. (2024) developed religion-centric hate speech corpora and re-trained Bangla-Bert-Base with an additional 159,367 offensive texts to create the hatebnBERT model, which outperformed baseline models but lacking advanced preprocessing (RG1), hybridization (RG2), and validation (RG3).

A few studies addressed multilingual settings, including Bengali. Nandi et al. (2024) combined mBERT and IndicBERT using stacking for Bengali, Marathi, and Hindi hate speech detection, achieving F1-scores of 92.30% (Bengali) and 81.50% (Marathi), yet omitted contextual preprocessing (RG1) and validation (RG3). Ghosh and Senapati (2024) evaluated transformer models across five Indian languages (including Bengali), where fine-tuned MuRIL-BERT reached 90.95% accuracy, but none of the RG1-RG3 factors were addressed. Ranasinghe and Zampieri (2021) tested XLM-R with transfer learning for seven languages, including Bengali, distinguishing covert and overt aggression while demonstrating cross-lingual adaptability but lacking preprocessing (RG1) and validation (RG3).

In summary, to our knowledge, only a few existing studies on Bengali cyberbullying detection have systematically examined the impact of additional preprocessing strategies, proposed robust transformer-based hybrid methods, or employed statistical testing to validate model effectiveness. Moreover, critical aspects such as scalability and adaptability remain largely unexplored, leaving ample scope to improve classification performance. This study addresses all these gaps by presenting a comprehensive framework for the effective classification of Bengali cyberbullying texts (see Table 1).

## 3 The transformer-stacking framework

**Figure 1:**
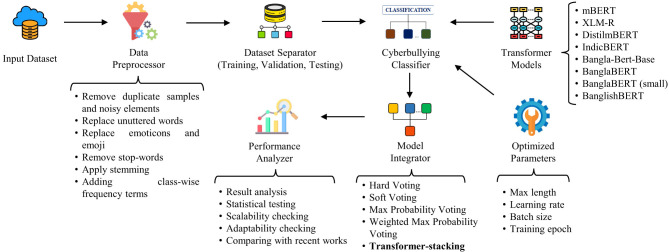
Overview of the *Transformer-stacking* framework.

Our *Transformer-stacking* framework combines enhanced preprocessing strategies with the integration of multiple high-performing transformer models using a stacking mechanism. This section outlines the development process of the proposed Bengali cyberbullying detection technique. As illustrated in Figure 1, the framework begins with a Bengali cyberbullying dataset as input. We then apply both general and advanced preprocessing operations to clean and structure the data. Next, eight SOTA transformer models are employed, each fine-tuned through hyperparameter optimization. The best-performing models are subsequently integrated using several transformer-based ensemble methods, among which the stacking ensemble is selected due to its ability to learn non-linear inter-model dependencies through a meta-learner. Finally, a comprehensive evaluation is conducted to assess the performance of the proposed framework. The following subsections describe each component of the framework in detail.

### 3.1 Input dataset

This study utilizes a publicly available Bengali cyberbullying dataset sourced from Mendeley Data (Ahmed et al., 2021a). The selection is based on three main factors: (i) its frequent use in recent research published in reputable journals (Aurpa et al., 2022; Akhter et al., 2023) and conferences (Wahid and Al Imran, 2023; Hoque and Seddiqui, 2023), (ii) its substantial size of 44,001 annotated entries, and (iii) its fine-grained labeling in five distinct categories of cyberbullying.

The dataset comprises Facebook comments directed at celebrities and includes the following fields: *comment* (the text of the comment), *category* (occupation of the celebrity), *gender* (male/female), *reacts* (number of likes/reactions), and *label* (target class). For this study, we consider only the *comment* and *label* fields. The *label* column annotates each sample into one of five classes: *Not Bully, Sexual, Troll, Religious*, and *Threat*.

**Figure 2:**
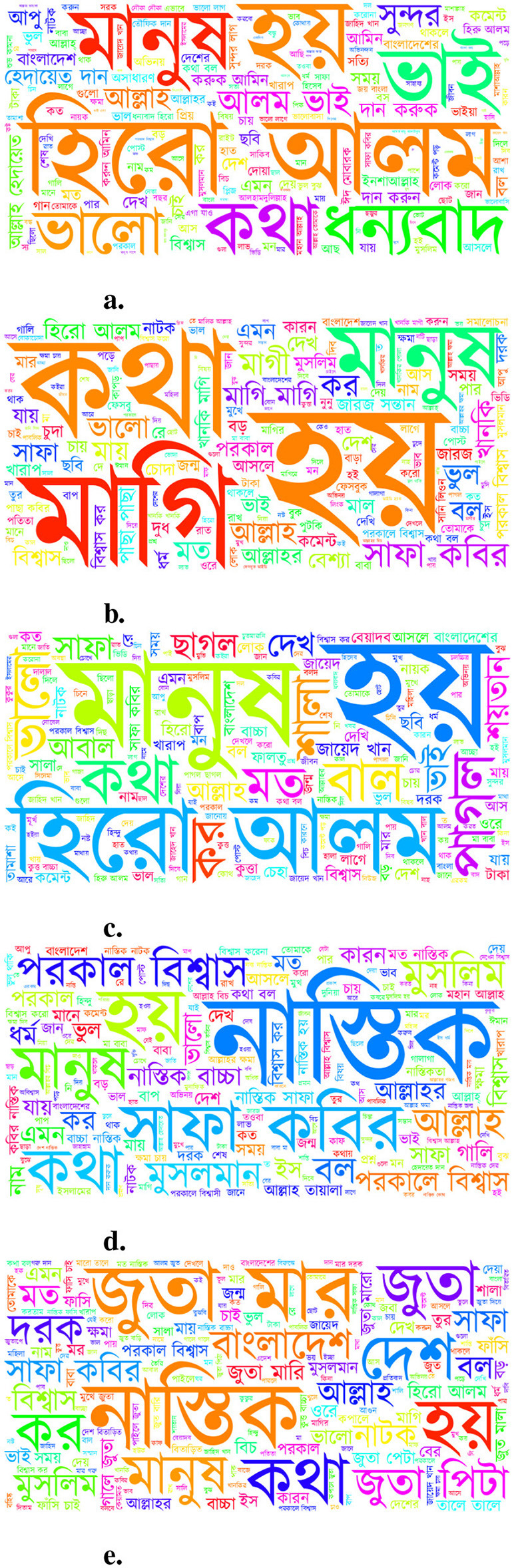
Word clouds showing frequent terms in each Bengali cyberbullying class. **(a)** Not Bully. **(b)** Sexual. **(c)** Troll. **(d)** Religious. **(e)** Threat.

Figure 2 shows the class-wise word clouds, revealing distinct lexical patterns across categories. For instance, positive expressions like “
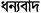
” (thanks) and “
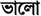
” (good) are prominent in the *Not Bully* class. In contrast, abusive terms such as “
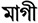
” (slut) and “
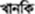
” (whore) dominate the *Sexual* class, while “
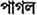
” (mad) and “
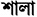
” (a mild profanity) appear frequently in the *Troll* class. Words like “
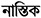
” (atheist) and “
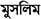
” (Muslim) are common in the *Religious* class, and violent expressions such as “
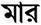
” (beat) and “
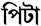
” (hit) are prevalent in the *Threat* category. However, some terms appear in multiple classes, indicating contextual ambiguity. For example, the term “
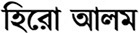
” (a celebrity name) frequently occurs in both the *Not Bully* and *Troll* classes, reflecting varying user intent across different posts.

**Figure 3:**
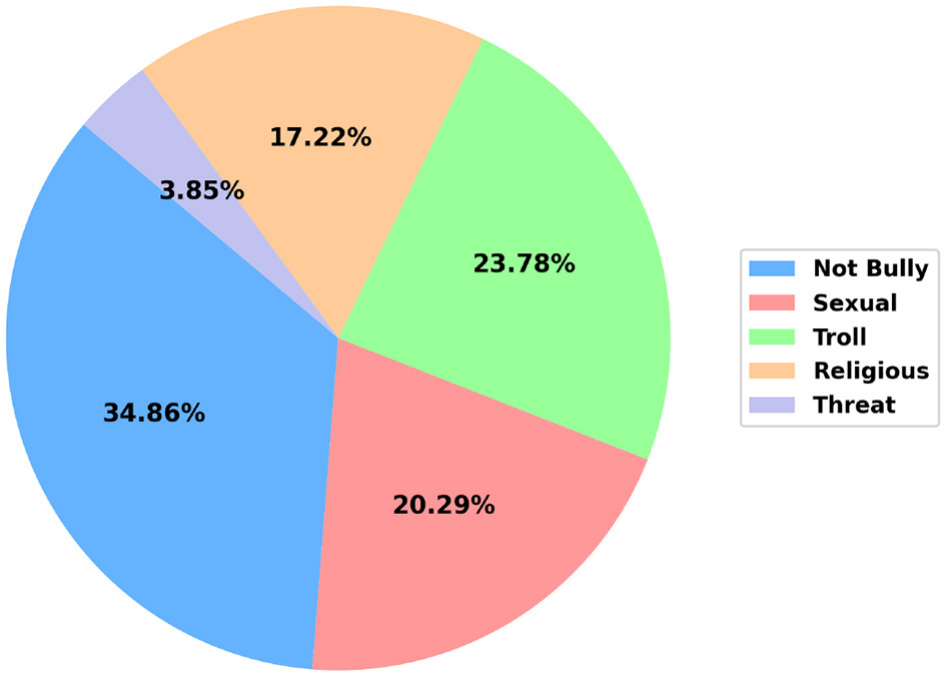
The percentage of comments across each cyberbullying category.

A detailed definition and an example for each class are presented in Table 2. Figure 3 visualizes the distribution of samples in the five categories. The dataset is imbalanced, with the *Not Bully* class having the highest number of samples (15,340) and the *Threat* class having the fewest (1,694).

**Table 2:** Interpretation of cyberbullying classes.

**Class label**	**Description**	**Example**
Not Bully	The comments that do not contain intentional attack to harass an individual	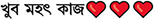 (Great work 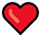 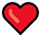 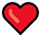 .)
Sexual	The Sexual class consists of user comments that propagate gender hatred towards an individual.	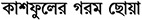 (The erotic touch of catkin.)
Troll	The Troll class comprises user comments that contain intentional mocks to insult another person.	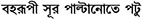 (A meme can be multi-faced.)
Religious	The comments contain offensive language and promote hostility towards specific religious groups.	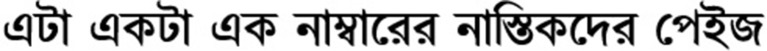 (This is a genuine atheist page.)
Threat	The user comments that contain explicit threats to hurt or kill another individual.	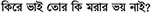 (Hello brother, you are not afraid to die?)

This study addresses two text classification tasks for this dataset: a binary classification task that determines whether a comment constitutes bullying or not, and a multiclass classification task that categorizes each comment into one of five specific cyberbullying classes—*Not Bully, Sexual, Troll, Religious*, and *Threat*. For the binary classification task, the four bullying categories (*Sexual, Troll, Religious*, and *Threat*) are grouped as the positive class, while *Not Bully* is treated as the negative class. Throughout the remainder of this paper, the binary classification task is referred to as *Sub-task A*, and the multiclass classification task as *Sub-task B*.

### 3.2 Data pre-processor

The raw dataset contains substantial noise, including punctuation marks, URLs, digits, and special characters (Asad et al., 2014). To address this, we first perform general preprocessing steps, such as removing HTML tags and punctuation, to remove irrelevant elements. We refer to this initial stage as **P**re**P**rocessing **C**ategory *PPC 1*. In addition to this, we introduce five more preprocessing categories designed to enrich the feature space and improve the classification of cyberbullying content. Each preprocessing category is described in detail below.

**Figure 4:**
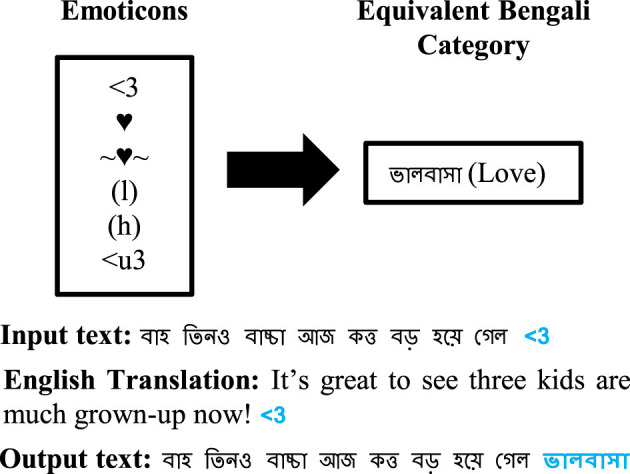
Replacing emoticon with a Bengali word.

**Figure 5:**
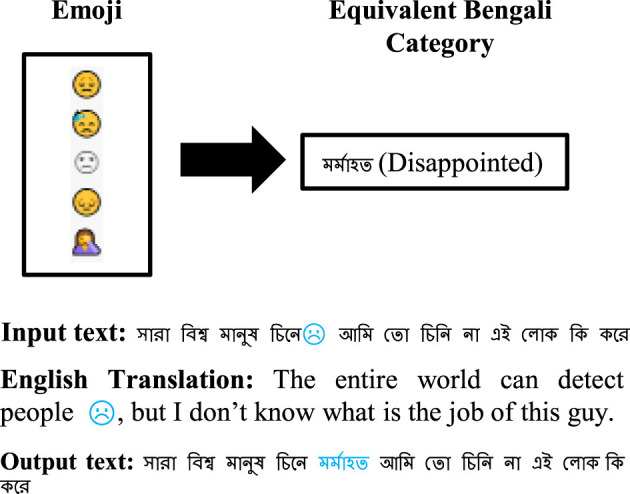
Replacing emoji with a Bengali word.

- **General preprocessing (*PPC 1*):** This category comprises eight essential preprocessing operations aimed at cleaning the dataset by removing irrelevant or noisy elements. These steps include: removing duplicate entries, eliminating thin-space Unicode characters (U+200C), correcting misplaced spaces around delimiters and sentence-ending symbols, stripping HTML tags, filtering out URLs, removing special characters and punctuation, eliminating digits, and discarding non-Bengali text.
- **Replacing censored or unuttered terms (*PPC 2*):** Many entries contain censored or masked abusive words using “*” characters (e.g., “
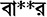
”). Our empirical observations show that these often carry negative sentiment. We replace such unuttered terms with a standardized Bengali token “
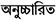
” (unuttered). This replacement is performed after correcting space misplacements.
- **Mapping emoticons and emojis to generalized Bengali sentiment expressions (*PPC 3*):** Emoticons (ASCII symbols) and emojis (Unicode characters) often express nuanced sentiments. We compiled two separate dictionaries: 402 emoticons grouped into 67 categories and 282 emojis grouped into 216 categories, each mapped to appropriate generalized Bengali words. For instance, happy-face emoticons are translated to “
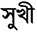
” (happy). Sample conversions are illustrated in Figures 4, 5. This preprocessing category is applied after URLs removal.
- **Removing stop-words and stemming (*PPC 4* and** ***PPC 5*):** These two standard preprocessing techniques are widely used in NLP to reduce feature dimensionality (Mahmud et al., 2014). However, some researchers argue that stop-word removal and stemming may discard useful features relevant for cyberbullying detection (Kumar et al., 2021). We empirically assess both inclusion and exclusion scenarios for these operations after removing non-Bengali text. This study employs a rule-based stemming technique specifically designed for Bengali text processing (Mahmud et al., 2014).
- **Injecting class-specific feature terms (*PPC 6*):** This is the final preprocessing step, where we incorporate class-indicative feature words into each text sample based on the presence of unique words associated with specific cyberbullying categories. We begin by constructing five dictionaries, one for each target class, by extracting distinct words from the entire dataset. An initial filtering removes terms with any of the following properties: (i) meaningless tokens (e.g., “
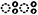
”), (ii) concatenated words without proper spacing (e.g., “
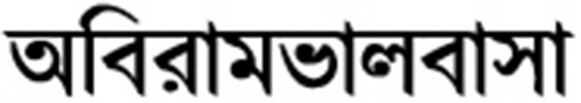
”), and (iii) words irrelevant to the semantic characteristics of their respective class (e.g., “
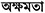
” (inability) is excluded from the *Religious* class). After this filtering, we address words that appear in multiple classes. Through empirical analysis, we retain each word in the class where it shows the strongest association and remove it from the others. We also account for dialectal variations, transliterated English words in Bengali, and minor spelling inconsistencies during this process. As a result, we obtain five refined dictionaries with unique word counts: 4726 for *Not Bully*, 740 for *Sexual*, 682 for *Troll*, 243 for *Religious*, and 85 for *Threat*. Next, we design three class-specific feature tokens to reflect the frequency of class-related words in each sample (see Supplementary material). For instance, in the *Not Bully* class, the features are: “
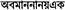
” (Not Bully once), “
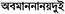
” (Not Bully twice), and “
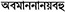
” (Not Bully more). Depending on how many class-specific words are detected in a sample, the corresponding token is prepended. For example, given the input text “
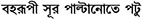
” (A meme can be multi-faced), which includes the Troll-class word “
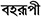
” (meme), the transformed text becomes “
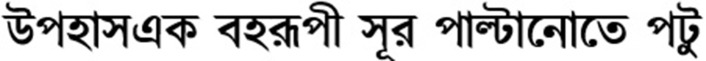
”. The core idea behind *PPC 6* is to inject explicit word-level class cues into the data, thereby guiding the model toward more accurate cyberbullying class identification.

To our knowledge, the three advanced preprocessing techniques: replacing censored or unuttered terms (*PPC 2*), mapping emoticons and emojis to generalized Bengali sentiment expressions (*PPC 3*), and injecting class-specific feature terms (*PPC 6*), have not been previously explored in Bengali cyberbullying detection. Their individual and combined impact on classification performance is analyzed in detail in Section 4.8.

### 3.3 Feature representation

This study employs eight pre-trained transformer models for feature extraction: XLM-R-base, IndicBERT, mBERT, DistilmBERT, Bangla-Bert-Base, BanglishBERT, BanglaBERT (small), and BanglaBERT. These models were selected to capture a diverse range of linguistic characteristics, including multilingual generalization, regional adaptability, and Bengali-specific language representation, thereby ensuring comprehensive feature extraction across both standard and informal Bengali texts. The XLM-R-base and IndicBERT utilize the SentencePiece Model (SPM) (Kudo and Richardson, 2018) for subword segmentation, incorporating both byte-pair encoding (BPE) (Sennrich et al., 2016) and unigram language modeling (Kudo, 2018). The remaining six models apply the WordPiece tokenization technique (Wu et al., 2016).

Each model is built with its own vocabulary and tokenization scheme, which leads to differences in the generated subword tokens, even when processing the same input. Notably, BanglaBERT and BanglaBERT (small) share an identical vocabulary, resulting in equivalent token sequences for any given input. A tokenization example using these models is provided in Supplementary material. During tokenization, all models incorporate special tokens, such as classification tokens ([CLS] or < s>), separator tokens ([SEP] or < /s>), and mask tokens ([MASK] or < MASK>), depending on the model architecture.

**Figure 6:**
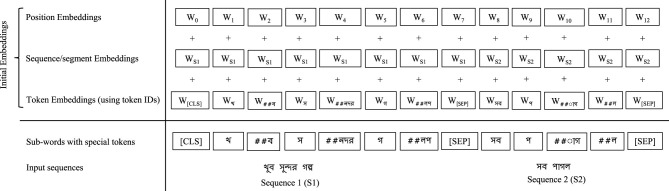
Initial embeddings of a transformer model (mBERT).

Each model follows the BERT-style embedding strategy to generate initial input representations. As shown in Figure 6, the initial embedding for a sequence is constructed by summing three components: token embeddings (representing subword tokens), segment embeddings (indicating sample index or sentence partition), and position embeddings (capturing the position of each token in the input sequence). These composite embeddings are then fed into the encoding layers of the respective transformer models for further processing.

### 3.4 Transformer models

We utilize eight transformer models: mBERT, DistilmBert, XLM-R-base, IndicBERT, Bangla-Bert-Base, BanglaBERT, BanglaBERT (small), and BanglishBERT that exhibit better performance in the Bengali NLP-related text classification tasks (Hoque et al., 2024a,b; Hoque and Salma, 2023; Chatterjee et al., 2023). The first four are multilingual pre-trained models that include Bengali text data in their pre-training stage, the subsequent three are Bengali language-specific pre-trained models, and the last is a bilingual model pre-trained on both Bengali and English data. These eight encoder-based transform models come from the original BERT model. To classify Bengali cyberbullying text using a BERT-based transformer model, firstly texts are tokenized (see Supplementary material) and then converted into vector forms (see Figure 6). These vectors, known as initial embeddings, are then passed into the transformer encoder blocks. Each encoder block has four layers: multi-head attention (MHA), first add & norm, feed-forward (FF), and second add & norm. The MHA layer takes the initial embedding in the form of three matrices: query (*Q*), key (*K*), and value (*V*). The MHA contains several self-attention layers, with each self-attention calculated using Equation 1 and the overall MHA computed using Equation 2 (Vaswani et al., 2017). In the first add & norm layer, the initial embedding matrix is added as a residual connection to the output matrix of the MHA, followed by layer normalization for stable training. Subsequently, single FF layer processes the result of the layer normalization. Finally, in the second add & norm layer, layer normalization is applied again over the sum of the first layer normalization result and the FF layer output. The transformer encoder blocks generate the final embeddings of each token. The linear layer receives the outcome of the [CLS] token, applies the softmax function, and predicts the most probable cyberbullying class by calculating the errors for the input text.


$$\begin{array}{l} \text{Attention}\left(Q,K,V\right)=\text{softmax}\left(\frac{QK^{T}}{\sqrt{d_{k}}}\right)V \end{array}$$ (1)

Where *d*_*k*_ denotes the size of *Q* and *K*, and $\frac{1}{\sqrt{d_{k}}}$ points out the scaling factor.


$$\begin{array}{r} \text{MultiHead}(Q,K,V)=\text{Concat}({\text{head}}_{1},...,{\text{head}}_{h})W^{O}\\ {\text{head}}_{i}=\text{Attention}(QW_{i}^{\;Q},KW_{i}^{\;K},VW_{i}^{\;V}) \end{array}$$ (2)

Here *h* is the number of attention heads, *W*^*O*^ is the output weight of the attention unit, and $W_{i}^{\;Q}$, $W_{i}^{\;K}$, and $W_{i}^{\;V}$ represent the *i*^*th*^ attention weight of *Q*, *K*, and *V* matrices, respectively.

BERT-based transformer models vary in architectures and model sizes (see Supplementary material). For further details, the following segments discuss each model in depth.

*Multilingual BERT:* BERT (Devlin et al., 2018) model has demonstrated superior performance in context-specific downstream tasks like question answering and language inference, outperforming other pre-trained models, such as Embeddings from Linguistic Models (ELMo) (Peters et al., 2018) and OpenAI Generative Pre-training (GPT) (Radford et al., 2018). Multilingual BERT (mBERT) follows the same principles as the original BERT but is pre-trained in 104 languages rather than one (English) (Pires et al., 2019). It employs pre-training and fine-tuning frameworks, utilizing a multi-head self-attention mechanism. The pre-training framework uses Masked Language Model (MLM) and Next Sentence Prediction (NSP) tasks to provide contextual comprehension of various languages. In contrast, the fine-tuning framework adjusts the model architecture to handle specific downstream tasks like sentence prediction and sentiment classification.

*XLM-RoBERTa:* XLM-RoBERTa (XLM-R) is a multilingual pre-trained transformer-based model designed to enhance cross-lingual and low-resource language comprehension through extensive training on over two terabytes of data from 100 languages, including Bengali (Conneau et al., 2020). It employs the SPM tokenization method with a vocabulary size of 250k, significantly higher than mBERT's 110k. XLM-R uses multi-head self-attention and excels in tasks like question answering and cross-lingual classification, outperforming mBERT, especially for low-resource languages.

*DistilmBERT:* DistilmBERT is a compressed version of mBERT that utilizes knowledge distillation (Buciluǎ et al., 2006; Hinton et al., 2015) to reduce the model size while retaining 97% of its language understanding ability (Sanh et al., 2019). It achieves this by removing the token-type embeddings and the pooler portions and halving the number of encoder layers. As a result, DistilmBERT is 40% smaller and trains 60% faster than mBERT.

*IndicBERT:* It follows the ALBERT (Lan et al., 2019) architecture and is pre-trained in twelve Indian languages, including Bengali (Kakwani et al., 2020). While maintaining BERT's basic architecture, IndicBERT introduces three key design changes:

- *Factorized embedding parameterization:* Let V, E, and H represent vocabulary, embedding, and hidden layer size, respectively. The embedding matrix (V × E) generates numerous embedding parameters when E ≡ H because of a higher value of V (e.g., 30K for BERT-base). IndicBERT factorizes this embedding matrix by decomposing it into two smaller matrices: (V × E) and (E × H), where E indicates a lower embedding dimension than H. Thus, the embedding parameters are minimized from O(V × H) to O(V × E + E × H). This optimization strategy is most effective when H ≫ E.
- *Cross-layer parameter sharing:* IndicBERT shares parameters across all the layers, as the FF layer shares parameters with other FF layers, and the MHA layer shares with other MHA layers to improve parameter efficiency (Kakwani et al., 2020).
- *Inter-sentence coherence loss:* BERT's NSP objective deals with topic and coherence predictions. Since the MLM objective nearly covers topic prediction, IndicBERT introduces the sentence order prediction (SOP) objective instead of NSP to emphasize inter-sentence coherence prediction (Kakwani et al., 2020). The SOP objective checks whether two sentences are in the correct order (positive example) or inverse order (negative example).

*Bangla-Bert-Base:* This transformer model, specific to the Bengali language, is based on the BERT architecture and was introduced by Sarker (2020). Pre-trained on two Bengali corpora (Bengali Common Crawl from OSCAR and Bengali Wikipedia), it outperforms other pre-trained models like mBERT in various NLP tasks, including sentiment analysis and hate speech detection.

*BanglaBERT:* This is another Bengali language-focused transformer model, pre-trained on more than 27.5 GB of data sourced from 110 prominent Bengali websites (Bhattacharjee et al., 2022). It results from pre-training the ELECTRA (Clark et al., 2020) model. In the pre-training phase, the model trains two neural network blocks: generator (*G*) and discriminator (*D*). Each block contains a transformer encoder for mapping a sequence of input tokens *x* = [*x*_1_, ..., *x*_*n*_] into a sequence of contextual embeddings vectors *h*(*x*) = [*h*_1_, ..., *h*_*n*_]. The generator predicts the probability of producing a token *x*_*t*_ at position *t* using a softmax layer, as shown in Equation 3.


$$\begin{array}{l} p_{G}\left(x_{t}|x\right)=\exp\left(e{\left(x_{t}\right)}^{T}h_{G}{\left(x\right)}_{t}\right)/\underset{x^{\prime }}{\sum }\exp\left(e{\left(x^{\prime }\right)}^{T}h_{G}{\left(x\right)}_{t}\right) \end{array}$$ (3)

Where *e* represents token embeddings. The discriminator determines the realness of the token *x*_*t*_ at position t in a way that this token comes from the original sequence or the generator's preferred distribution. In this case, the discriminator utilizes the sigmoid layer using Equation 4:


$$\begin{array}{l} D\left(x,t\right)=\text{sigmoid}\left(w^{T}h_{D}{\left(x\right)}_{t}\right) \end{array}$$ (4)

**Figure 7:**
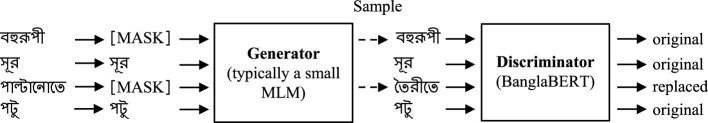
Masked token replacement process of the BanglaBERT model.

Figure 7 illustrates the masked token replacement process through the two blocks—the generator and the discriminator. The generator performs the MLM objective. This objective randomly selects a set of positions from an input sequence *x* = [*x*_1_, ..., *x*_*n*_] to mask out *m* = [*m*_1_, ..., *m*_*k*_], and replaces the tokens of those positions with the token ([MASK]) using Equation 5. The generator is then trained to gain the ability to guess the appropriate identities of the masked-out tokens. These predicted tokens replace the mask tokens. A variable *x*^*crpt*^ stores these predicted tokens using Equation 6. The discriminator block utilizes the Replaced Token Detection task rather than BERT's NSP task to learn about the *x*^*crpt*^ tokens and whether they are real or fake by comparing them with input x. The model inputs can be formally written as:


$$\begin{array}{r} m_{i}\sim \text{unif}\left\{1,n\right\},\;\text{for }i=1\text{ to }k\\ x^{\text{masked}}=\text{REPLACE}\left(x,m,\left[\text{MASK}\right]\right) \end{array}$$ (5)

$$\begin{array}{r} \hat{x_{i}}\sim p_{G}\left(x_{t}|x^{\text{masked}}\right),\;\text{for }i\in m\\ x^{\text{crpt}}=\text{REPLACE}\left(x,m,\hat{x}\right) \end{array}$$ (6)

Additionally, the loss functions can be calculated using Equations 7, 8:


$$\begin{array}{l} L_{\text{MLM}}(x,\theta_{G})=𝔼(\sum_{i\in m}-\log p_{G}(x_{t}|x^{\text{masked}})) \end{array}$$ (7)

$$\begin{array}{l} L_{\text{Disc}}(x,\theta_{D})=𝔼(\sum_{t=1}^{n}-𝟙(x_{t}^{\text{crpt}}=x_{t})\log D(x^{\text{crpt}},t)\\ \;-𝟙(x_{t}^{\text{crpt}}\neq x_{t})\log(1-D(x^{\text{crpt}},t))) \end{array}$$ (8)

The generator uses maximum likelihood during the training phase. The combined loss is optimized over a large corpus (X) using the following formula:


$$\begin{array}{l} \underset{\theta_{G},\theta_{D}}{\min}\underset{x\in X}{\sum }L_{\text{MLM}}\left(x,\theta_{G}\right)+L_{\text{Disc}}\left(x,\theta_{D}\right) \end{array}$$

BanglaBERT discards the generator after pre-training and fine-tunes the discriminator for downstream tasks, achieving superior performance in Bengali Natural Language Understanding tasks, such as Sentiment Classification and Natural Language Inference (Bhattacharjee et al., 2022).

*BanglaBERT (small):* It follows the same pre-training procedure as Bangla-BERT but is a lighter version with four attention heads instead of twelve (Bhattacharjee et al., 2022). This reduces the model size by minimizing embedding (*E*), hidden (*H*), and feed-forward layer (*H*_*ff*_) dimensions. Consequently, it takes less time to pre-train and fine-tune than BanglaBERT.

*BanglishBERT:* It is pre-trained in Bengali and English, following the BanglaBERT architecture (Bhattacharjee et al., 2022). With a vocabulary size of about 16k for each language, it excels in zero-shot cross-lingual transfer, showing better performance in many Bengali NLP-related tasks.

### 3.5 Transformer ensemble

The ensemble technique aims to combine multiple transformer models to achieve higher predictive performance than any single model (Dietterich, 2000). This study investigates five ensemble approaches—*Hard Voting, Soft Voting, Max Probability Voting, Weighted Max Probability Voting*, and *Transformer-stacking*—applied across eight transformer architectures. Each method is briefly outlined below.

#### 3.5.1 Hard voting

In hard voting, each transformer predicts a class label, and the final label ŷ is determined by majority voting as shown in Equation 9:


$$\begin{array}{l} \hat{y}=\arg\underset{c\in C}{\max}\overset{M}{\underset{m=1}{\sum }}𝕀(y^{\left(m\right)}=c), \end{array}$$ (9)

where C is the set of classes, *M* is the number of models, *y*^(*m*)^ is the *m*-th model's predicted label, and *I*(·) is the indicator function.

#### 3.5.2 Soft voting

Soft voting averages the predicted class probabilities from all models and selects the class with the highest mean probability, as given in Equation 10.


$$\begin{array}{l} \hat{y}=\arg\underset{c\in C}{\max}\frac{1}{M}\overset{M}{\underset{m=1}{\sum }}P^{\left(m\right)}\left(c\right), \end{array}$$ (10)

where *P*^(*m*)^(*c*) denotes the probability assigned to class *c* by model *m*.

#### 3.5.3 Max probability voting

This method selects the class corresponding to the single highest predicted probability across all models, as shown in Equation 11.


$$\begin{array}{l} \hat{y}=\arg\underset{c\in C}{\max}\underset{m\in \left\{1,\ldots ,M\right\}}{\max}P^{\left(m\right)}\left(c\right). \end{array}$$ (11)

#### 3.5.4 Weighted max probability voting

To prioritize stronger models, weighted max probability voting multiplies each model's probability by its normalized accuracy score *w*_*m*_, where $\overset{M}{\underset{m=1}{\sum }}w_{m}=1$, as given in Equation 12.


$$\begin{array}{l} \hat{y}=\arg\underset{c\in C}{\max}\underset{m\in \left\{1,\ldots ,M\right\}}{\max}[w_{m}\cdot P^{\left(m\right)}\left(c\right)]. \end{array}$$ (12)

#### 3.5.5 Transformer-stacking

The *Transformer-stacking* strategy adopts a stacking ensemble architecture, where multiple transformer models act as base learners. Each transformer is independently trained and produces class-level predictions on the validation data. These prediction outputs are then concatenated to create an aggregated feature representation.

This unified feature vector serves as the input to a meta-classifier, specifically, a multilayer perceptron (MLP), which is a feedforward neural network comprising one or more hidden layers. The MLP is trained to learn the optimal combination of the base models' outputs to improve classification performance.

**Figure 8:**
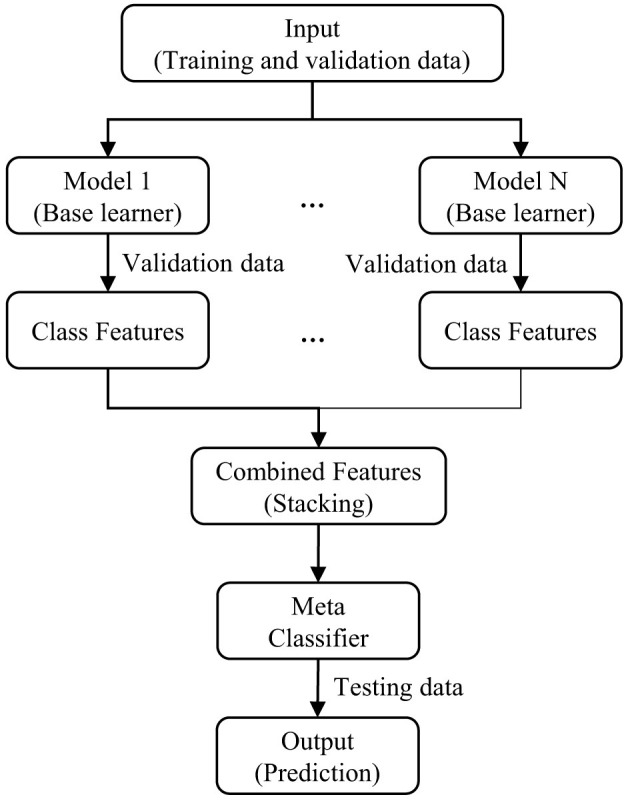
A schematic diagram of the *Transformer-stacking* ensemble.

After training, the meta-classifier is employed to predict the final class labels for unseen test instances. This two-tiered architecture effectively captures diverse predictive signals from multiple transformer models, leveraging their complementary strengths. A schematic overview of the *Transformer-stacking* ensemble is illustrated in Figure 8.

**Rationale for design choices:** The selection of XLM-R-base, mBERT, and Bangla-Bert-Base as base learners is motivated by their complementary linguistic coverage and architectural diversity. XLM-R-base, trained on a massive multilingual corpus, provides deep cross-lingual representations beneficial for handling code-mixed and diverse Bengali content. mBERT contributes robustness against subword-level noise and informal expressions due to its WordPiece tokenization across 104 languages. Bangla-Bert-Base, a monolingual model trained exclusively on Bengali corpora, excels in capturing fine-grained syntactic and semantic nuances specific to the language. By integrating these three models, the ensemble leverages both multilingual generalization and monolingual precision.

The choice of stacking as the ensemble approach, rather than voting or averaging, is based on its ability to learn non-linear inter-model dependencies through a secondary learner. An MLP is used as the meta-classifier due to its capacity to approximate complex mappings between the base model outputs and the true class labels. This design enables the framework to adaptively weight the contribution of each transformer, leading to improved generalization and robustness across both balanced and imbalanced datasets.

## 4 Experimental evaluation

This section provides a comprehensive evaluation of the proposed Bengali cyberbullying detection framework. It begins by outlining the experimental setup, including hardware specifications and platform configurations for binary and multiclass classification tasks (Section 4.1). This is followed by a discussion of hyperparameter tuning, where four key parameters are optimized to maximize model performance (Section 4.2). The final implementation setup is then introduced, in which the optimized models and a hybrid method are applied using a specified dataset configuration (Section 4.3). Subsequently, the section presents the impact of additional preprocessing strategies on classification performance (Section 4.4). Classification outcomes from individual transformer models and the proposed *Transformer-stacking* framework are then reported (Section 4.5) and compared against the recent SOTA approaches (Section 4.6). A class-wise performance breakdown highlights category-specific strengths and weaknesses (Section 4.7). Next, the section analyzes both the positive and negative impacts of additional preprocessing operations on the classification of cyberbullying (Section 4.8). In addition, it delves into a multidimensional evaluation of the proposed framework (Section 4.9), covering statistical significance tests (e.g., McNemar's test) (Section 4.9.1), benchmarking against baseline models (Section 4.9.2), assessing scalability and adaptability (Section 4.9.3), and error analysis to identify common misclassification trends (Section 4.9.4). Together, these evaluations offer a holistic view of the effectiveness, generalizability, and potential areas for improvement of the proposed framework.

### 4.1 Experimental setup

This experiment utilizes a Bengali cyberbullying dataset for both binary and multiclass text classification tasks, leveraging eight pre-trained transformer-based models. Given the high computational demands, the experiments are conducted on Google Colab's cloud platform, which provides access to an NVIDIA Tesla T4 GPU with 15 GB of RAM, along with a Jupyter Notebook environment preloaded with essential Python libraries and packages (Carneiro et al., 2018).

### 4.2 Hyper-parameter tuning

To achieve optimal model performance, we experimented with four key hyperparameters, informed by general-purpose preprocessing operations from the *PPC 1* group (see Section 3.2). The parameters and their value ranges, described in Table 3, were selected based on empirical studies and within the constraints of our hardware specifications. The dataset was initially split into training (90%) and validation (10%) sets to tune these parameters. Using the Ktrain Python library proposed by Maiya (2022), we conducted extensive experiments to determine the best settings for each model, as detailed in Table 4.

**Table 3:** Hyperparameter overview.

**Parameter name**	**Data type**	**Description**	**Value**
Max token length (*maxLen*)	Integer	Maximum number of tokens for each comment.	Minimum value = 100, Maximum value = 192
Learning rate (*lr*)	Float	It adjusts the rate at which a loss function approaches the convergence of the curves.	Value = [1e-05, 2e-05, 3e-05, 4e-05, 5e-05, 6e-05]
Epochs (*epoch*)	Integer	The number of times with which the whole training set is utilized for learning the model.	Minimum value = 2, Maximum value = 10
Batch size (*batchSize*)	Integer	The number of comments going through in each iteration of every epoch throughout model training.	Minimum value = 12, Maximum value = 40

**Table 4:** Optimal hyperparameter settings of the used models.

**Model**	*max*−*Len*	*lr*	*epoch*	*batch*−*Size*	**Validation loss**
mBERT	160	5e-05	4	20	0.422
XLM-R-base	160	4e-05	4	20	0.398
DistilmBERT	160	5e-05	3	24	0.474
IndicBERT	160	4e-05	4	12	0.543
Bangla-Bert-Base	160	5e-05	3	28	0.449
BanglaBERT	160	5e-05	3	16	0.476
BanglaBERT (small)	160	4e-05	4	16	0.494
BanglishBERT	160	4e-05	3	16	0.459

### 4.3 Final implementation setup

After adjusting the hyperparameters, this study moves on to the final use of the transformer models and their combined methods to carry out both *Sub-task A* (binary classification) and *Sub-task B* (multiclass classification). The dataset is partitioned into training (70%), validation (15%), and testing (15%) sets to ensure a rigorous and reliable evaluation. The goal of this work is to develop an effective cyberbullying detection framework for Bengali text through systematic model optimization and the use of high-performance computing resources.

### 4.4 Results: additional preprocessing operations

This study evaluates the effect of five additional preprocessing categories, *PPC 2* through *PPC 6*, on classification performance. Accordingly, six experimental configurations, denoted as *EC 1* to *EC 6*, are designed and tested for both *Sub-task A* and *Sub-task B*. Each configuration corresponds to a specific preprocessing category: *EC 1* includes only the general preprocessing operations (*PPC 1*), while *EC 2* to *EC 6* combine *PPC 1* with one of the additional preprocessing categories (*PPC 2* through *PPC 6*, respectively).

The outcomes of these experiments are presented in Table 5. From the results, it is evident that three preprocessing categories, *PPC 2, PPC 3*, and *PPC 6*, consistently improve classification performance across both subtasks. In contrast, *PPC 4* and *PPC 5* lead to a decrease in accuracy.

**Table 5:** Performance results in terms of accuracy (%) for every experimental configuration.

**Method**	**EC 1**	**EC 2**	**EC 3**	**EC 4**	**EC 5**	**EC 6**
**Sub-task A**						
mBERT	91.34	91.37 ↑	91.39 ↑	90.49 ↓	91.14 ↓	92.68 ↑
XLM-R-base	91.29	91.46 ↑	91.52 ↑	91.25 ↓	91.10 ↓	93.10 ↑
DistilmBERT	89.96	89.98 ↑	90.02 ↑	89.28 ↓	89.52 ↓	91.36 ↑
IndicBERT	88.57	88.59 ↑	88.71 ↑	88.17 ↓	88.48 ↓	90.44 ↑
Bangla-Bert-Base	90.44	90.71 ↑	90.77 ↑	90.23 ↓	90.32 ↓	92.33 ↑
BanglaBERT	90.87	90.96 ↑	90.94 ↑	90.56 ↓	90.45 ↓	91.97 ↑
BanglaBERT (small)	88.88	88.98 ↑	89.24 ↑	88.56 ↓	88.77 ↓	90.74 ↑
BanglishBERT	90.67	90.80 ↑	90.73 ↑	89.85 ↓	90.16 ↓	92.09 ↑
**Sub-task B**						
mBERT	85.65	85.73 ↑	86.03 ↑	85.52 ↓	85.73 ↑	87.76 ↑
XLM-R-base	86.18	86.85 ↑	86.40 ↑	85.17 ↓	85.68 ↓	88.02 ↑
DistilmBERT	83.61	83.80 ↑	83.63 ↑	83.13 ↓	83.22 ↓	85.25 ↑
IndicBERT	80.88	81.40 ↑	81.09 ↑	80.05 ↓	80.72 ↓	83.54 ↑
Bangla-Bert-Base	84.53	84.58 ↑	84.58 ↑	84.01 ↓	84.47 ↓	86.46 ↑
BanglaBERT	84.68	84.84 ↑	84.82 ↑	83.28 ↓	84.21 ↓	86.03 ↑
BanglaBERT (small)	82.82	82.97 ↑	83.08 ↑	81.71 ↓	81.95 ↓	84.29 ↑
BanglishBERT	84.67	85.08 ↑	84.82 ↑	83.82 ↓	84.26 ↓	86.44 ↑

*EC 1* = *PPC 1* + Transformer model, *EC 2* = *PPC 1* + *PPC 2* + Transformer model, *EC 3* = *PPC 1* + *PPC 3* + Transformer model, *EC 4* = *PPC 1* + *PPC 4* + Transformer Model, *EC 5* = *PPC 1* + *PPC 5* + Transformer model, *EC 6* = *PPC 1* + *PPC 6* + Transformer Model, Upward arrow (↑) = Positive impact with respect to the baseline approach (*EC 1*), and Downward arrow (↓) = Negative impact with respect to the baseline approach (*EC 1*).

These findings empirically validate the effectiveness of the preprocessing techniques described in *PPC 2, PPC 3*, and *PPC 6* (refer to Section 3.2). Among these, *PPC 6* yields the most significant performance gain, followed by *PPC 3*, which generally outperforms *PPC 2*. On the other hand, *PPC 4* tends to degrade performance more severely than *PPC 5*.

A detailed discussion on the influence of these preprocessing operations is provided in Section 4.8. The following section presents the performance results of the transformer models when the three most effective preprocessing techniques are applied in combination.

### 4.5 Results: combined preprocessing and transformer models

Building on the promising results from *EC 2, EC 3*, and *EC 6*, the new experimental category, *EC 7*, combines the corresponding preprocessing categories, *PPC 1, PPC 2, PPC 3*, and *PPC 6*. All eight transformer models are evaluated under this new configuration. Table 6 presents the outcomes of this experiment in terms of precision, recall, F1-score, and accuracy. A comparison between *EC 7* and *EC 6* (see Table 5) reveals that nearly all models demonstrate improved accuracy in *EC 7*, with the sole exception of BanglaBERT (small) in *Sub-task B*, which exhibits a slight decline. These findings validate that the combined application of *PPC 2, PPC 3*, and *PPC 6*, along with the baseline preprocessing (*PPC 1*), effectively enhances classification performance.

**Table 6:** Performance of transformer models for combined preprocessing in *EC 7*.

**Model**	**Sub-task A**				**Sub-task B**			
	*P* **(%)**	*R* **(%)**	*F* **(%)**	*A* **(%)**	*P* **(%)**	*R* **(%)**	*F* **(%)**	*A* **(%)**
**Individual transformer model**								
mBERT	93.04	92.99	93.01	92.99	87.77	87.79	87.74	87.79
XLM-R-base	93.23	93.22	93.22	93.22	88.06	88.07	88.05	88.07
DistilmBERT	91.76	91.78	91.77	91.78	85.63	85.54	85.51	85.54
IndicBERT	91.22	91.23	91.23	91.23	83.62	83.61	83.58	83.61
Bangla-Bert-Base	92.41	92.43	92.42	92.43	86.83	86.86	86.83	86.86
BanglaBERT	92.12	92.06	92.08	92.06	86.10	86.11	86.08	86.11
BanglaBERT (small)	90.87	90.77	90.81	90.77	84.09	84.16	84.07	84.16
BanglishBERT	92.12	92.11	92.11	92.11	86.99	86.95	86.95	86.95
**Transformer ensemble**								
Hard Voting	93.58	93.57	93.58	93.57	88.94	88.94	88.91	88.94
Soft Voting	93.55	93.54	93.55	93.54	88.96	88.95	88.93	88.95
Max Probability Voting	93.47	93.47	93.47	93.47	88.68	88.69	88.65	88.69
Weighted Max Probability Voting	93.47	93.47	93.47	93.47	88.69	88.71	88.67	88.71
Transformer-stacking	**93.60**	**93.62**	**93.61**	**93.62**	**89.28**	**89.23**	**89.23**	**89.23**

*P* = Precision; *R* = Recall; *F* = F1-score; *A* = Accuracy.

Among the eight transformer models, XLM-R-base consistently achieves the highest performance in both tasks. In *Sub-task A*, it attains a precision of 93.23%, recall of 93.22%, F1-score of 93.22%, and accuracy of 93.22%. For *Sub-task B*, its performance remains strong with precision, recall, F1-score, and accuracy of 88.06%, 88.07%, 88.05%, and 88.07%, respectively. mBERT ranks second, yielding 93.04% precision, 92.99% recall, 93.01% F1-score, and 92.99% accuracy in *Sub-task A*, and 87.77%, 87.79%, 87.74%, and 87.79% across the same metrics in *Sub-task B*. Bangla-Bert-Base secures the third-best performance, achieving 92.41% precision, 92.43% recall, 92.42% F1-score, and 92.43% accuracy in *Sub-task A*, and 86.83%, 86.86%, 86.83%, and 86.86% in *Sub-task B*. The remaining five models rank in the following order based on their accuracy in *Sub-task A*: BanglishBERT, BanglaBERT, DistilBERT, IndicBERT, and BanglaBERT (small). A similar trend is observed for *Sub-task B*, with the only difference being that IndicBERT and BanglaBERT (small) exchange positions.

Since IndicBERT and BanglaBERT (small) exhibit lower performance than the other six transformer models, the ensemble experiments are conducted using the top six models across all five ensemble strategies (see Section 3.5). For each technique, various model combinations of sizes ranging from two to six are evaluated. The optimal combinations for every ensemble method are summarized in Table 6.

Interestingly, the combination of the top three models: XLM-R-base, mBERT, and Bangla-Bert-Base, consistently produces the best results across all ensemble techniques. Although all ensembles improve precision, recall, F1-score, and accuracy compared to individual models, the proposed *Transformer-stacking* approach, employing three base learners and an MLP as the meta-classifier, outperforms the other four ensemble methods in both *Sub-task A* and *Sub-task B*.

In *Sub-task A*, the *Transformer-stacking* method achieves a precision of 93.60%, recall of 93.62%, F1-score of 93.61%, and accuracy of 93.62%. For *Sub-task B*, it reaches 89.28% precision, 89.23% recall, 89.23% F1-score, and 89.23% accuracy. The *Hard Voting* and *Soft Voting* ensembles demonstrate comparable results, with accuracies of 93.57% and 93.54% in *Sub-task A*, and 88.94% and 88.95% in *Sub-task B*, respectively. Meanwhile, *Max Probability Voting* and *Weighted Max Probability Voting* yield slightly lower accuracies, 93.47% in *Sub-task A* and 88.69% and 88.71% in *Sub-task B*, respectively.

Given that our framework integrates three robust preprocessing operations and the *Transformer-stacking* strategy, we henceforth refer to this method as our proposed approach for the remainder of this paper. A more detailed analysis of its performance and behavior is presented in the subsequent sections.

### 4.6 Results: performance comparison with state-of-the-art methods

We identified the seven SOTA studies that utilized the same Bengali cyberbullying dataset (Ahmed et al., 2021a). One of these studies (Akhter et al., 2023) employed the IHT technique, which resulted in the exclusion of a large portion of the dataset, removing 35,531 out of 44,001 samples. Since IHT filters out misclassified or challenging instances (Smith et al., 2014), this significantly alters the dataset's composition. Therefore, we excluded this study from our comparative analysis to maintain fairness and consistency.

Table 7 provides a comparative overview of our framework's performance against the remaining recent works. Except for Ahmed et al. (2021b), all other studies focused solely on *Sub-task B*. The study in Ahmed et al. (2021b) achieved an F1-score of 82.00% and an accuracy of 87.91% in *Sub-task A* using a CNN-LSTM hybrid model. For *Sub-task B*, several studies, including Ahmed et al. (2021b), Aurpa et al. (2022), and Emon et al. (2022), achieved 85.00% accuracy using ensemble methods with SVM, BERT-base, and XLM-R-base, respectively. Wahid and Al Imran (2023) reported improved results using a multi-feature transformer-based deep learning model, obtaining an F1-score of 86.00% and accuracy of 86.30%. More recently, Hoque and Seddiqui (2023) and Hoque and Seddiqui (2024b) applied transformer-based ensemble strategies with hard and soft voting, achieving accuracies of 87.54% and 87.61%, respectively.

**Table 7:** Performance comparison between *Transformer-stacking* and recent related works on the Bengali cyberbullying dataset (Ahmed et al., 2021a).

**References**	**Approach**	**Sub-task A**		**Sub-task B**	
		*F* **(%)**	*A* **(%)**	*F* **(%)**	*A* **(%)**
Ahmed et al. (2021b)	CNN-LSTM	82.00	87.91	-	-
	Ensemble with SVM	-	-	84.00	85.00
Aurpa et al. (2022)	BERT-base	-	-	83.04	85.00
Emon et al. (2022)	XLM-R-base	-	-	86.00	85.00
Wahid and Al Imran (2023)	Multi-feature transformer-based DL method	-	-	86.00	86.30
Hoque and Seddiqui (2023)	Transformer-ensemble (hard voting)	-	-	87.52	87.54
Hoque and Seddiqui (2024b)	Transformer-ensemble (soft voting)	-	-	87.59	87.61
Our proposed framework	Transformer-stacking	**93.61**	**93.62**	**89.23**	**89.23**

*F* = F1-score; *A* = Accuracy.

In contrast, our proposed *Transformer-stacking* framework, which integrates three impactful preprocessing strategies along with an effective stacking ensemble architecture, achieves superior results in both subtasks. It records an F1-score of 93.61% and an accuracy of 93.62% in *Sub-task A* and an accuracy of 89.23% and an F1-score of 89.23% in *Sub-task B*, thereby outperforming all previously published approaches on this dataset. Thus, it delivers a 5.69% accuracy improvement in *Sub-task A* and accuracy gains of 1.85%–4.97% in *Sub-task B*.

### 4.7 Results: class-wise performance of transformer-stacking

**Figure 9:**
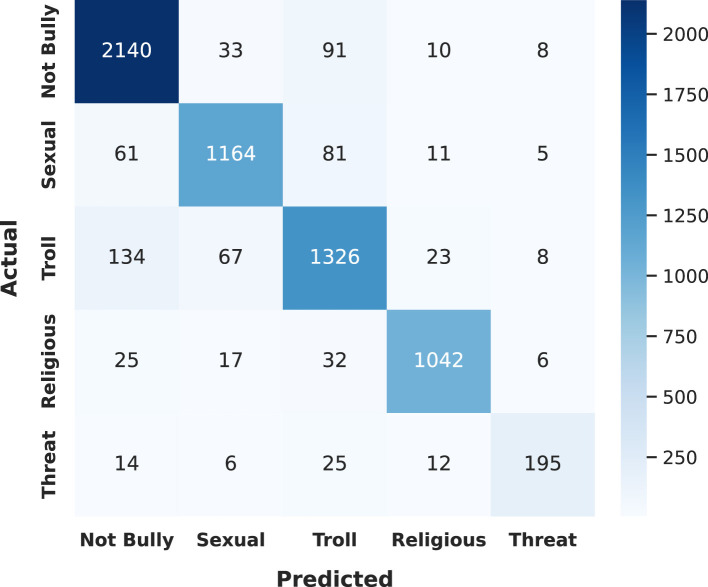
Confusion matrix of the proposed *Transformer-stacking* framework on *Sub-task B*.

Since *Sub-task B* involves multiclass classification across five distinct cyberbullying categories, including *Not Bully, Sexual, Troll, Religious*, and *Threat*, we focus our class-wise performance analysis on this task. Table 8 and Figure 9 present the performance of the proposed *Transformer-stacking* framework across these classes.

**Table 8:** Class-level performance metrics of *Transformer-stacking* on *Sub-task B*.

**Class**	**Pricision (%)**	**Recall (%)**	**F1-score (%)**
Not Bully	90.92	92.11	91.51
Sexual	88.85	88.05	88.45
Troll	83.32	85.62	84.46
Religious	94.72	92.78	937.4
Threat	89.25	75.79	81.97

The framework demonstrates strong performance on the *Not Bully* class, achieving an F1-score of 91.51%. This can be attributed to the class's large representation in the dataset (34.86%), which enables the model to learn its patterns effectively. Similarly, this framework performs exceptionally well on the *Religious* class, with a high F1-score of 93.74%. Empirical analysis reveals that samples in this class frequently contain distinctive class-specific keywords, allowing for more accurate classification.

For the *Sexual* class, the proposed framework yields moderate results, with a precision of 88.85%, recall of 88.05%, and an F1-score of 88.45%. The slightly lower recall indicates a higher number of false negatives (FN), where true positive instances were incorrectly classified as other categories. Specifically, 88 and 56 *Sexual* samples were misclassified as *Troll* and *Not Bully*, respectively.

Performance for the *Troll* class is comparatively weaker, with a precision of 83.32%, recall of 85.62%, and F1-score of 84.46%. Our proposed framework produces a high number of false positives (FP)—instances where non-*Troll* samples are incorrectly classified as *Troll*. Specifically, 117 *Not Bully* and 88 *Sexual* samples are misclassified as belonging to the *Troll* class. Additionally, the class suffers from a high number of FNs, with 121 and 76 actual *Troll* instances incorrectly labeled as *Not Bully* and *Sexual*, respectively. This overlap highlights a strong correlation and potential semantic similarity between the *Troll* and *Not Bully* classes, as reflected in the confusion matrix (see Figure 9).

The weakest performance is observed for the *Threat* class, with an F1-score of 81.97%. This is largely due to the class's underrepresentation in the dataset (only 3.85%), which limits the model's ability to learn meaningful patterns. The recall for this class drops to 75.79%, with 28, 14, and 13 *Threat* samples misclassified as *Troll, Religious*, and *Not Bully*, respectively.

In summary, the *Transformer-stacking* framework excels in identifying well-represented and semantically distinct classes, but performance degrades for minority and semantically overlapping categories, suggesting opportunities for further improvement in future research work.

### 4.8 Discussion: impact of additional preprocessing

Tables 5, 6 present the impact of five additional preprocessing components: *PPC 2* (*EC 2*), *PPC 3* (*EC 3*), *PPC 4* (*EC 4*), *PPC 5* (*EC 5*), and *PPC 6* (*EC 6*), on classification performance. Among them, *PPC 2, PPC 3*, and *PPC 6* contribute positively to model performance, whereas *PPC 4* and *PPC 5* result in decreased accuracy.

The *PPC 2* component replaces censored or masked offensive words with the Bengali placeholder term “
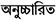
” (unuttered), as detailed in Section 3.2. These words are often associated with profanity or abuse. This substitution helps the model better learn patterns related to cyberbullying. For instance, a text such as “
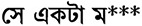
” (a sexually offensive phrase) is transformed into “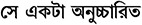,” where the added token “
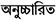
” helps identify the instance as belonging to the *Sexual* class.

*PPC 3* maps emoticons and emojis to equivalent generalized Bengali expressions, thus preserving the emotional or semantic content of the text (see Section 3.2). Since emojis often carry sentiment, their mapping enhances model understanding. For example, in “
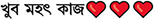
” (Great work
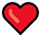

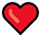

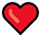
), each heart symbol is replaced with “
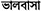
” (love), reinforcing the *Not Bully* classification. This improves both contextual understanding and sentiment recognition.

*PPC 6* introduces synthetic class-indicative feature words to guide the model, especially when few class-specific keywords exist in a sample. For example, in “
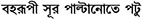
” (He is a master of disguise), a sample from the *Troll* class, *PPC 6* prepends the word “
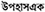
” (troll-one), which reinforces the association with the *Troll* class. This boosts model sensitivity to weak signals during training.

In contrast, *PPC 4* and *PPC 5* degrade classification performance. These components reduce sentence length by removing stopwords or non-informative tokens, which inadvertently eliminate contextually important words. For instance, the original *Not Bully* sample “
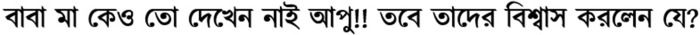
” is reduced to “
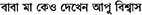
” after applying *PPC 4*, stripping away crucial linguistic cues. As a result, transformer models such as mBERT incorrectly label the text as *Troll*.

Furthermore, the Bengali stemmer from Mahmud et al. (2014) used in *PPC 5* occasionally produces errors. It may generate unknown or incorrect stems such as 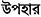 (gift) → 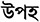, or 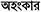 (pride) → 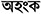. In other cases, it alters meanings entirely, e.g., 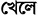 (play) → 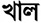 (canal), or 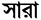 (whole) → 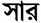 (fertilizer). These inaccuracies reduce semantic consistency and hinder model performance.

In summary, careful selection and design of preprocessing steps, particularly those that enrich semantic representation without distorting the original context, can significantly enhance cyberbullying detection in Bengali text.

### 4.9 Discussion: impact of transformer-stacking

The *Transformer-stacking* framework proposed in this study has been rigorously evaluated across multiple experimental dimensions to assess its effectiveness in Bengali cyberbullying detection. This section synthesizes the findings from four critical perspectives: statistical testing, internal model comparison, scalability and adaptability justification, and error analysis. The following subsections elaborate on each of these aspects in detail.

#### 4.9.1 Statistical comparison using McNemar's test

To perform a rigorous statistical comparison between our proposed *Transformer-stacking* framework and eight baseline transformer models, we employed McNemar's test on both *Sub-task A* and *Sub-task B* (see Table 9). This test evaluates the statistical significance of performance differences by comparing the number of instances misclassified differently by two models, allowing for robust pairwise significance testing on classification outputs.

**Table 9:** McNemar's test results comparing *Transformer-stacking* with individual transformer models for Bengali cyberbullying classification.

**Model**	**Sub-task A**			**Sub-task B**		
	**Statistic**	**p-value**	**Significance**	**Statistic**	**p-value**	**Significance**
mBERT	8.466	0.004	Significant	28.639	≈0	Significant
XLM-R-base	3.592	0.058	Not Significant	24.671	≈0	Significant
DistilmBERT	38.691	≈0	Significant	114.513	≈0	Significant
IndicBERT	58.598	≈0	Significant	208.980	≈0	Significant
Bangla-Bert-Base	22.804	≈0	Significant	67.184	≈0	Significant
BanglaBERT	24.404	≈0	Significant	74.926	≈0	Significant
BanglaBERT (small)	78.859	≈0	Significant	170.958	≈0	Significant
BanglishBERT	27.053	≈0	Significant	58.788	≈0	Significant

*p* < 0.05 ⇒ Significant, *p*≥0.05 ⇒ Not significant.

For *Sub-task A*, among the eight comparisons, seven models show a statistically significant difference (*p* < 0.05) when compared with *Transformer-stacking*, indicating that our proposed framework performs significantly better than these models. Notably, DistilmBERT, IndicBERT, and BanglaBERT (small) demonstrate very high test statistics (38.69, 58.60, and 78.86, respectively), emphasizing substantial disagreement in misclassified instances. XLM-R-base yields a *p*-value of 0.058 in comparison with *Transformer-stacking*, narrowly missing the conventional threshold for statistical significance. This suggests that while both models perform similarly in the binary task, *Transformer-stacking* may still offer a marginal advantage.

In contrast, for *Sub-task B*, all eight baseline models yield statistically significant differences (*p* < 0.05) when compared with *Transformer-stacking*. The test statistics are markedly higher than those in Sub-task A, particularly for IndicBERT (208.98), BanglaBERT (small) (170.96), and DistilmBERT (114.51), implying that *Transformer-stacking* substantially improves multiclass classification accuracy. Even models that were not significantly different in *Sub-task A*, such as XLM-R-base, are found to be significantly outperformed by *Transformer-stacking* in *Sub-task B*.

In summary, the McNemar's test results underscore the robustness and generalization capability of *Transformer-stacking*. While the binary classification task shows only one non-significant comparison, the multiclass task reveals consistent and statistically significant superiority of the proposed framework across all baselines. This further suggests that *Transformer-stacking* is particularly well-suited for handling nuanced distinctions between multiple categories of Bengali cyberbullying.

#### 4.9.2 Performance comparison with transformer models and ensemble methods

Table 6 demonstrates that the proposed *Transformer-stacking* framework, augmented with three additional preprocessing components (*PPC 2, PPC 3*, and *PPC 6*), consistently outperforms each of the eight individual transformer models and four ensemble methods in the Bengali cyberbullying classification task. The impact of these preprocessing strategies is elaborated in Section 4.8.

Among the individual models incorporated into the *Transformer-stacking* framework, XLM-R-base, mBERT, and Bangla-Bert-Base achieve notably higher classification accuracy due to their complementary representational capabilities. XLM-R-base, pre-trained on a massive 2.5TB multilingual CommonCrawl corpus spanning 100 languages, including Bengali (Conneau et al., 2020; Liu, 2019), captures deep cross-lingual semantics through its robust SentencePiece tokenizer. mBERT, trained on Wikipedia data from 104 languages, leverages WordPiece tokenization to remain resilient against subword-level noise and informal expressions typical of social media content (Pires et al., 2019; Devlin et al., 2018). Conversely, Bangla-Bert-Base, trained solely on extensive Bengali corpora (Sarker, 2020), excels in grasping the syntactic and semantic subtleties of standard Bengali. This diversity among the base learners ensures coverage across formal, informal, and code-mixed contexts, critical for detecting cyberbullying language variation. Further qualitative validation is provided in Table 10, which presents real-world examples where the *Transformer-stacking* framework produces more accurate predictions than individual models.

**Table 10:** Qualitative justification of the *Transformer-stacking* framework using selected test samples.

**Cyberbullying text**	**Predicted Labels**									**Gold label**
	**mBERT**	**Distill-mBERT**	**XLM-R-base**	**Indic-BERT**	**Bangla-bert-base**	**Bangla-BERT**	**Bangla-BERT (small)**	**Banglish-BERT**	**Trans-former-stacking**	
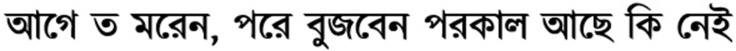 (First you die, then you will understand whether there is an afterlife or not.)	0	3	4	4	4	3	3	0	4	4
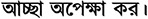 (Well, wait.)	4	0	0	0	0	0	4	4	0	0
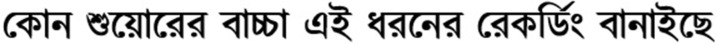 (What rascal is making such recordings?)	2	0	0	0	0	2	2	2	2	2

Not Bully ⇒ 0, Sexual ⇒ 1, Troll ⇒ 2, Religious ⇒ 3, Threat ⇒ 4.

While ensemble techniques such as *Hard Voting, Soft Voting, Max Probability Voting*, and *Weighted Max Probability Voting* enhance robustness by aggregating multiple transformer predictions, their aggregation is typically static. For instance, voting-based methods assign either equal or fixed weights to model outputs, ignoring inter-model dependencies or contextual nuances among base predictions. As a result, these techniques fail to exploit complex non-linear relationships between model confidence distributions—especially when transformers exhibit complementary error patterns across different bullying categories or linguistic variations.

The *Transformer-stacking* framework, on the other hand, introduces a dynamic learning layer via a meta-classifier, specifically a multilayer perceptron (MLP). The MLP is trained on the concatenated output probabilities from the three top-performing transformers (XLM-R-base, mBERT, and Bangla-Bert-Base), allowing it to learn non-linear mappings that better capture inter-model interactions. In essence, the meta-classifier learns how to emphasize the strengths of each base model—such as mBERT's resilience to noise, Bangla-Bert-Base's syntactic precision, and XLM-R's contextual generalization, depending on the linguistic characteristics of each instance. This adaptive fusion mechanism significantly improves the model's ability to generalize across diverse online discourse.

Empirical evidence supports this observation: the proposed *Transformer-stacking* achieves the highest accuracy of 93.62% for *Sub-task A* and 89.23% for *Sub-task B*, surpassing all other ensemble approaches by a margin of 0.15–0.55 percentage points. Notably, the performance gain in *Sub-task B*, which involves multi-class classification, highlights the MLP's effectiveness in discriminating subtle inter-class differences, something that fixed-weight ensembles often fail to capture. For example, the text “
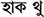
” (Spit on you.) was misclassified as *Not Bully* by both the *Hard Voting* and *Soft Voting* ensembles, while another instance, “
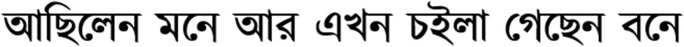
” (She was on everyone's mind, but now she's gone off into the wild), was also misclassified as *Not Bully* by the *Max Probability Voting* and *Weighted Max Probability Voting* methods. In contrast, the proposed *Transformer-stacking* framework correctly identifies both instances as belonging to the *Troll* category.

By strategically combining these three complementary base transformers and employing an adaptive MLP meta-classifier, the proposed *Transformer-stacking* framework effectively captures higher-order relationships between prediction patterns. Consequently, it achieves superior generalizability and robustness over both individual transformer models and other ensemble variants.

#### 4.9.3 Scalability and adaptability assessment

To further validate the scalability and adaptability of our proposed *Transformer-stacking* framework, we evaluate it on two additional Bengali datasets of varying sizes (to assess scalability) and different cyberbullying-related contexts (to assess adaptability). The first dataset, focused on hate speech, is sourced from Romim et al. (2021), while the second, centered on threats and abusive language, is obtained from Chakraborty and Seddiqui (2019).

The hate speech dataset contains 30,000 samples, with 10,000 labeled as hate speech (class 1) and the remaining 20,000 as non-hate speech (class 0). The second dataset is comparatively smaller, comprising 5,644 samples with an approximately balanced class distribution; about 50% of the samples are considered threats or abusive, and the rest are non-abusive. The inclusion of datasets with varying sizes highlights the scalability and adaptability of the proposed framework.

Following the experimental protocols of the original studies, we split both datasets into training, validation, and test sets. Across both corpora, our proposed *Transformer-stacking* framework consistently outperforms existing approaches.

For the hate speech dataset, Romim et al. (2021) achieved an F1-score of 91.10% and an accuracy of 87.50% using an SVM-based approach. More recently, Ghosh and Senapati (2024) utilized the MuRIL-BERT model, reporting an F1-score of 90.98% and an accuracy of 90.95%. In contrast, our proposed *Transformer-stacking* framework surpasses both, achieving an F1-score of 91.45% and a notably higher accuracy of 91.42%.

For the threat and abusive dataset, Chakraborty and Seddiqui (2019) report an accuracy of 78.00%. A more recent study by Hoque and Seddiqui (2024a) improves the performance using an mBERT-based technique, achieving 80.17% accuracy and a 77.70% F1-score. In contrast, our *Transformer-stacking* framework achieves the highest results, with an F1-score of 83.40% and an accuracy of 83.47%.

Table 11 summarizes the comparative performance of *Transformer-stacking* against existing methods on both datasets. These results underscore the scalability and adaptability of our framework for Bengali cyberbullying detection across diverse domains.

**Table 11:** Comparison of *Transformer-stacking* with existing methods on two additional Bengali cyberbullying datasets.

**Dataset**	**Author**	**Method**	**F1-score**	**Accuracy**
Hate Speech (Romim et al., 2021)	Romim et al., 2021	SVM	91.10	87.50
	Ghosh and Senapati, 2024	MuRIL-BERT	90.98	90.95
	**Our proposed framework**	**Transformer-stacking**	**91.45**	**91.42**
Threat and Abusive (Chakraborty and Seddiqui, 2019)	Chakraborty and Seddiqui, 2019	SVM	-	78.00
	Hoque and Seddiqui, 2024a	mBERT	77.70	80.17
	**Our proposed framework**	**Transformer-stacking**	**83.40**	**83.47**

#### 4.9.4 Analysis of misclassifications and model limitations

The *Transformer-stacking* framework faces challenges in accurately distinguishing between the *Not Bully* and *Troll* classes, primarily due to semantic overlap. For instance, the comment “
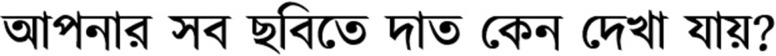
” (Why are your teeth visible in all of your pictures?), which belongs to the *Troll* class, is incorrectly predicted as *Not Bully*. This reflects the contextual ambiguity that often exists between neutral and sarcastic expressions.

Furthermore, the model struggles to correctly identify samples from the *Threat* class due to its relatively small representation in the dataset. For example, the threatening text “
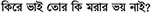
” (Hey brother, are you not afraid of dying?) is misclassified as *Troll*, indicating limited learning on minority class characteristics.

Additionally, the effectiveness of the three auxiliary preprocessing techniques, *PPC 2* (unuttered word replacement), *PPC 3* (emoji and emoticon mapping), and *PPC 6* (injection of class-specific feature words), is inherently dependent on the presence of their respective textual elements. When a comment does not contain emojis, unuttered or censored words, or class-indicative lexical patterns, these preprocessings have no impact on the input representation, thus offering no added value to classification performance in such cases.

Another contributing factor to misclassification is the noisy nature of user-generated content, which often includes unstructured syntax, misspellings, grammatical inconsistencies, and code-mixing with regional dialects. These linguistic complexities reduce the model's ability to encode meaningful representations.

## 5 Conclusion

This study presents an effective transformer-based ensemble, *Transformer-stacking*, for Bengali cyberbullying detection. The framework combines three high-performing transformer models, XLM-R-base, mBERT, and Bangla-Bert-Base, using a stacking ensemble strategy, where a multi-layer perceptron classifier is employed as the meta-learner. This architecture is further enhanced with targeted preprocessing techniques tailored to the characteristics of cyberbullying texts, including replacing censored or unuttered terms, mapping emoticons and emojis to generalized Bengali sentiment expressions, and injecting class-specific feature terms. Comprehensive experiments show that these enhancements significantly boost classification performance on a widely used Bengali cyberbullying dataset. The proposed framework achieves an F1-score of 93.61% and accuracy of 93.62% in binary classification (*Sub-task A*), and an F1-score and accuracy of 89.23% in multiclass classification (*Sub-task B*), outperforming all eight baseline transformer models, four ensemble methods, and recent state-of-the-art approaches. Notably, it delivers a 5.69% accuracy improvement in *Sub-task A* and accuracy gains of 1.85%–4.97% in *Sub-task B*. Statistical validation using McNemar's test confirms the significance of these improvements. In addition, evaluations on two external datasets demonstrate the scalability and adaptability of the framework. Error analysis highlights persistent challenges, such as class imbalance, label confusion, and noisy input. Overall, the *Transformer-stacking* framework offers a powerful, scalable, and adaptable solution for Bengali cyberbullying classification, representing a substantial advancement in online abuse detection for low-resource languages.

Future work will address semantic overlap among cyberbullying classes by incorporating richer contextual and user-level cues, while data augmentation and adaptive re-sampling will be explored to mitigate class imbalance in minority categories. Efforts will also focus on enhancing preprocessing adaptability to better handle linguistic noise, dialectal variations, and code-mixed text. Furthermore, we plan to extend the framework into ontology- and graph-based approaches for harasser identification and behavioral analysis, thereby integrating deep learning with semantic reasoning to strengthen contextual understanding of cyberbullying dynamics.

## Data Availability

Publicly available datasets were analyzed in this study. This data can be found here: Ahmed et al. (2021a).
