# Supplementary material for: Advancing cyberbullying detection in low-resource languages: a transformer- stacking framework for Bengali
Source: Front Artif Intell. 2026 Jan 13;8:1679962. doi: 10.3389/frai.2025.1679962 (PMC12835245; doi:10.3389/frai.2025.1679962)
Supplement: Supplementary file 1 [file Data_Sheet_1.pdf]

frontiers

Supplementary Material

1 CLASS-SPECIFIC FEATURES

Every cyberbullying class may connect with three additional feature words based on the number of occurrences of the class-wise unique words for each sample, as shown in Table S1.

Table S1. Feature words related to class-wise unique word count.

Class Label	Class-wise unique word count for each sample		
	**One**	**Two**	**More than two**
Not Bully	অবমাননানয়এক (Not Bully once)	অবমাননানয়দুই (Not Bully twice)	অবমাননানয়বহু (Not Bully more)
Sexual	লিঙ্গগতএক (Sexual once)	লিঙ্গগতদুই (Sexual twice)	লিঙ্গগতবহু (Sexual more)
Troll	উপহাসএক (Troll once)	উপহাসদুই (Troll twice)	উপহাসবহু (Troll more)
Religious	ধর্মীয়এক (Religious once)	ধর্মীয়দুই (Religious twice)	ধর্মীয়বহু (Religious more)
Threat	হুমকিএক (Threat once)	হুমকিদুই (Threat twice)	হুমকিবহু (Threat more)

2 TOKENIZATION OF BERT'S VARIANTS

A sample of tokenization with the tokenizing technique of eight transformer models for a given text is shown in Table S2.

Table S2. A tokenization example for an input text.

**Model**	**Tokenization Technique**	**Input Text:** বহরূপী সুর পাল্টানোতে পটু
		**Tokens with IDs (without special tokens)**
mBERT	WordPiece	'ব' (640), '##হ' (22512), '##রপ' (80300), '##ী' (13493), 'স' (648), '##র' (11276), 'প' (638), '##াল' (27085), '##টা' (33221), '##নো' (47583), '##তে' (15343), 'প' (638), '##ট' (16160)
DistilBert	WordPiece	'ব' (970), '##হ' (26145), '##র' (11128), '##ূপ' (83790), '##ী' (13100), 'স' (978), '##ূ' (111236), '##র' (11128), 'প' (968), '##াল' (28725), '##্ট' (33072), '##ান' (18770), '##ো' (16431), '##তে' (15613), 'প' (968), '##ট' (18513), '##ু' (16166)
Bangla-Bert-Base	WordPiece	'বহর' (15410), '##পী' (35672), 'সর' (15803), 'পালটা' (52116), '##নে' (6552), '##াতে' (16050), 'পট' (18453)
XLM-R-base	Sentence Piece Model	'__' (6), 'বহ' (98738), 'রূপ' (96538), 'ী' (4091), '__স' (12945), 'ূ' (25690), 'র' (999), '__' (6), 'পাল' (80181), '্ট' (56253), 'ানো'
IndicBERT	Sentence Piece Model	'__বহ' (72261), 'রূপ' (17256), 'ী' (551), '__' (8), 'সূর' (122803), '__পাল' (15333), 'টানো' (142871), 'তে' (362), '__প' (3821), 'টু' (19170)
BanglaBERT	WordPiece	'বহর' (22887), '##ূপ' (1402), '##ী' (428), 'সূর' (2873), 'পাল্টা' (6888), '##নো' (3351), '##তে' (773), 'পটু' (12164)
BanglaBERT (small)	WordPiece	'বহর' (22887), '##ূপ' (1402), '##ী' (428), 'সূর' (2873), 'পাল্টা' (6888), '##নো' (3351), '##তে' (773), 'পটু' (12164)
BanglishBERT	WordPiece	'বহ' (1341), '##রূপ' (3317), ''##ী'' (315), ''সূর'' (2519), 'পাল্টা' (6926), '##নো' (3044), '##তে' (576), 'পটু' (12213)

3 MODEL SIZE AND STRUCTURAL ARCHITECTURE OF TRANSFORMER MODELS

The structural architectures and sizes of the eight BERT model variants are shown in Table S3, where L denotes the number of transformer encoder layers, E the embedding size, H the hidden size, H_{ff} the hidden intermediate feed-forward layer size, A the number of attention heads, V the vocabulary size, $G2D$ the generator-to-discriminator ratio, and P the number of parameters.

Table S3. Transformer models' sizes and architectures.

**Models**	L	E	H	H_{ff}	A	V	$G2D$	P
mBERT	12	768	768	3072	12	110k	-	172M
XLM-R-base	12	768	768	3072	12	250k	-	270M
DistilmBERT	6	768	768	3072	12	120k	-	134M
IndicBERT	12	128	768	3072	12	200k	-	12M
Bangla-Bert-Base	12	768	768	3072	12	102k	-	110M
BanglaBERT	12	768	768	3072	12	32k	$\frac{1}{3}$	110M
BanglaBERT (small)	12	128	256	1024	4	32k	$\frac{1}{3}$	110M
Banglish-BERT	12	768	768	3072	12	32k	$\frac{1}{3}$	110M
